# The prognostic and predictive value of homologous recombination deficiency status in patients with advanced stage epithelial ovarian carcinoma after first-line platinum-based chemotherapy

**DOI:** 10.3389/fonc.2024.1372482

**Published:** 2024-06-10

**Authors:** Yiqing Xu, Yi-Ju Amy Chen, Yunhong Wu, Angela Saverimuthu, Archana Jadhav, Rehana Bhuiyan, Jason Sandler, Jiang Yio, Vivek Kumar

**Affiliations:** ^1^ Division of Hematologic Oncology, Department of Internal Medicine, Maimonides Medical Center, Brooklyn, NY, United States; ^2^ Department of Obstetrics & Gynecology, Division of Gynecologic Oncology, Weill Cornell Medical College and New York Presbyterian/Queens Hospital, Flushing, NY, United States; ^3^ Department of Biostatistics and Medical Informatics, University of Wisconsin-Madison, Madison, WI, United States; ^4^ Department of Medicine, Brigham and Women’s Hospital, Boston, MA, United States

**Keywords:** ovarian cancer, platinum-based chemotherapy, overall survival, DNA repair, homologous recombination, genetic testing

## Abstract

**Objective:**

Homologous recombination (HR) comprises series of interrelated pathways that repair double-stranded DNA breaks and inter-strand crosslinks. It provides support for DNA replication to recover stalled or broken replication forks. Compared with homologous recombination proficiency (HRP), cancers with homologous recombination deficiency (HRD) are more likely to undergo cell death when treated with DNA-damaging agents, such as platinum agents, and have better disease control.

**Methods:**

Patients diagnosed with stage III/IV ovarian cancer, early stages with recurrence, who received adjuvant chemotherapy after debulking surgery, and who also had known HR status were eligible.

**Results:**

Forty-four patients were included, with 21 in the HRD group (including 8 with germline mutations) and 23 in the HRP group. The HRD group was composed predominantly of serous carcinoma (95.2%), while mucinous (n=3) and clear cell (n=1) cases were all found in the HRP group. Stage III/IV disease was 66.7% and 91.3% in HRD and HRP groups, respectively (p=0.064). Patients who were optimally debulked to no residual disease was 90.0% and 72.7% (p=0.243), respectively. Late line use of PARP inhibitors was 33.3% and 17.4% (p=0.303). Median PFS was 22.5 months (95% CI, 18.5 - 66.6) and 21.5 months (95% CI, 18.3-39.5) (p=0.49) in HRD and HRP respectively. Median platinum free interval (PFI) was 15.8 months (95% CI 12.4-60.4) and 15.9 months (95% CI 8.3-34.1) (p=0.24), respectively. Median OS was 88.2 months (95% CI 71.2-NA) and 49.7 months (95% CI 35.1-NA) (p=0.21). The PFS of the patients with germline *BRCA* mutations (n=5) was 54.3 months (95% CI 23.1-NA) and 21.5 months (95% CI 18.3-39.5) in the HRP group (p=0.095); the PFI difference was 47.7 months (95% CI 17.6-NA) in the *BRCA* mutation group, and 15.9 months (95% CI 12.4-60.4) in HRP, showing statistical significance (p=0.039); while the median OS was NA and 49.7 months (95% CI 35.1-NA) respectively (p=0.051). When adding two additional patients with somatic *BRCA* mutations to the germline *BRCA* mutation carriers, the median OS is NA (95% CI 73, NA) versus 49.7 months (95% CI 35.1, NA) for HRP (p=0.045).

**Conclusions:**

HRD status was not associated with longer PFS or PFI in advanced ovarian cancer who received first line adjuvant platinum-based chemotherapy. Its role as a prognostic marker for overall survival is suggested, particularly in the subgroup with germline and somatic *BRCA* mutations.

## 1 Introduction

Ovarian cancer is the most common gynecologic malignancy, with 18518 new cases and 13438 deaths recorded in the United States in 2020 (1). About 65% of all epithelial ovarian carcinomas, and about 80% of serous ovarian carcinomas, are stage III or IV at the time of diagnosis (2). Unfortunately, stage IIIC ovarian carcinoma only carries a five-year survival rate of about 42%, a figure that drops to 26% for stage IV disease (2). The current standard treatment for advanced stage ovarian cancer is cytoreduction surgery followed by systemic platinum-based combination chemotherapy (3, 4), optimal cytoreduction to residual disease of 1cm or less improves survival (5). As about 70% of patients with stage III or IV disease still experience cancer recurrence after front-line treatment, intensive research over the years has explored various therapeutic approaches to improve disease control. Randomized studies evaluated the role of intraperitoneal (IP) chemotherapy (6, 7), dose-dense strategies with weekly paclitaxel (8–10), the addition of hyperthermic intraperitoneal chemotherapy to interval cytoreductive surgery (11), and incorporation of the vascular endothelial growth factor (VEGF) inhibitor, bevacizumab, into systemic treatment (12–15). Despite these tremendous efforts, these approaches has yielded limited improvements. The addition of bevacizumab improves PFS but not OS (12, 14) while dose-dense chemotherapy failed to improve PFS or OS significantly in the confirmatory GOG 0262 trial (8). Findings from IP chemotherapy studies showed mixed results. The GOG 252 trial, which compared intravenous (IV) versus two IV/IP chemotherapy regimens in combination with bevacizumab, showed that PFS was not significantly increased with either IP regimen when combined with bevacizumab (7). On the other hand, the iPocc study compared IV versus IP carboplatin in combination with dose-dense paclitaxel showed modest improve in PFS with IP therapy (16).

The development of a new class of medication, namely Poly (ADP-ribose) polymerase (PARP) inhibitors, has deepened our understanding of cellular DNA damage repair mechanisms in response to environmental insults and replication errors. PARP inhibitors act by trapping PARP1 and PARP2 proteins at existing single-stranded breaks in DNA strands, thus interfering with single-stranded DNA damage repair and eventually leading to the accumulation of double-stranded DNA breaks. These double-stranded breaks are repaired via homologous recombination (HR) (17), which is the most relevant set of DNA repair pathways in ovarian cancer (18). HR comprises a series of interrelated pathways that function to repair double-stranded DNA breaks and inter-strand crosslinks, and to provide support for DNA replication to recover stalled or broken replication forks (19).

While sensitivity to cisplatin treatment serves a predictive marker for PARP inhibitor activity, commercial assays to test homologous recombination deficiency have been developed and validated, mainly the FoundationOne CDx assay and the Myriad MyChoice CDx assay (20). The FoundationOne CDx assay (21) quantifies the loss of heterozygosity (LOH) or the presence of insertions and deletions, copy number alterations, gene rearrangements, etc., that frequently occur in HRD cells. The Myriad MyChoice CDx assay (22) examines the levels of loss of heterozygosity, telomeric allelic instability and large-scale state transitions (LST) and report a genomic instability score (GIS). The FoundationOne CDx assay was used in the ARIEL trials where rucaparib maintenance was examined after response to platinum therapy (23) for determination of HRD status. Maintenance therapy with PARP inhibitors has yielded significant improvement in PFS among *BRCA* 1/2 mutation carriers and HRD patients compared to the HRP patients, in both first-line and subsequent-line settings (24, 25). Therefore, HRD is a predictive marker for benefits from PARP inhibitor.

Platinum drugs are known to exert lethality by generating intra-strand adducts and inter-strand crosslinks that damage the structure of DNA, halting DNA synthesis and transcription (26). Such defects are repaired through HR in normal circumstances (27, 28). Sharing the same mechanism of HR for repair between platinum lethality and *BRCA 1/2* mutations and HRD status, the *BRCA* mutations and HRD status appear to predict platinum sensitivity. In multiple retrospective studies, ovarian cancer patients with BRCA1/2 mutations exhibit higher response rate to platinum chemotherapy with prolonged PFS and OS compared to those without mutations (29–31). Furthermore, in the report by Pennington et. al, patients with germline, or somatic *BRCA 1/2*, or other HR genes, namely *ATM, BARD1, BRIP1, CHEK1, CHEK2, FAM175A, MRE11A, NBN, PALB2, RAD51C* and *RAD51D*, also showed superior primary platinum sensitivity, defined as maintenance of complete response ≥ 6 months (31). In addition, superior PFS and OS was observed in *BRCA* mutation positive ovarian cancer patients after receiving IP cisplatin treatment in comparison to those who are *BRCA* mutations negative (32).

Lastly, studies have also shown that patients with *BRCA* mutations appear to have a longer overall survival, possibly associated with their superior response to platinum and non-platinum based treatments (31, 33–35).

Considering that the presence of HRD could augment the lethal effects of platinum-base chemotherapy due to deficiencies in DNA repair (36), we hypothesized that HRD ovarian cancers would have superior response to platinum-based treatment when compared with HRP ovarian cancers, and may exhibit longer progression-free survival (PFS), platinum-free intervals (PFI), as well as overall survival (OS) after first-line platinum-based chemotherapy.

## 2 Materials and methods

### 2.1 Study design

Ovarian cancer patients diagnosed at Maimonides Cancer Center from 1/1/2010 through 11/30/2020 with stage III/IV, or stage I or II with subsequent recurrence were eligible. Patient were required to have next gene sequencing (NGS) testing or germline testing with known germline mutations implicated in the ovarian cancer development. In all cases, the original tumor tissue was used for NGS testing. All NGS tests were performed at Foundation Medicine, Inc. (Cambridge, MA) using the FoundationOne CDx test (21). The formalin-fixed, paraffin-embedded (FFPE) ovarian tumor tissue was used for extraction of DNA. About 50-1000 ng of DNA will undergo whole-genome shotgun library construction and hybridization-based capture of all coding exons from 309 cancer-related genes, one promoter region, one non-coding (ncRNA), and selected intronic regions from 34 commonly rearranged genes, 21 of which also include the coding exons. Using the Illumina^®^ HiSeq 4000 platform, hybrid capture–selected libraries are sequenced to high uniform depth (targeting >500X median coverage with >99% of exons at coverage >100X). Sequence data is then processed using a customized analysis pipeline designed to detect all classes of genomic alterations, including base substitutions, indels, copy number alterations (amplifications and homozygous gene deletions), and selected genomic rearrangements (e.g., gene fusions).

HRD status was defined as a loss of heterozygosity (LOH) score ≥ 16 as determined by FoundationOne commercial testing. As the inclusion of the LOH score in characterization of HRD status was approved in New York State only in 2019, the LOH scores for the 31 cases that were tested before 2019 were obtained from Foundation Medicine, Inc. through a data transfer agreement for this research project. Seven patients had commercial test results. We also included six patients into the HRD group solely based on their positive germline mutations implicated in HR pathway, also employed by other studies (31, 37).

We excluded patients who had pure neuroendocrine/small cell pathology, who lacked treatment data, who had borderline ovarian tumor, or who had stage I or II disease with no recurrence during the study period.

Debulking status was defined as the following: optimal debulking to no gross residual disease; optimal debulking to residual disease less or equal to 1 cm; or suboptimal debulking with residual disease greater than 1 cm. Tumor mutational burden (TMB), reported in units of mutations per megabase (mut/Mb), was calculated by counting the total number of all synonymous and non-synonymous variants present at 5% allele frequency or greater (after filtering). The definitions of low, intermediate and high TMB levels were based on Foundation Medicine CDx reporting criteria. Low TMB corresponds to a TMB level of 0-5 mut/Mb, intermediate TMB represents 6-15 mut/Mb, and high TMB indicates values of 16 mut/Mb or higher.

PFS measured the time from the date of cancer diagnosis to the date of first CT imaging recurrence (or to the detection of elevated CA-125 followed by treatment, if CT imaging was not performed). Platinum-free interval (PFI) measured the time from the date of the final cycle of first-line (adjuvant) platinum chemotherapy to the date of first disease recurrence. OS measured the time from date of cancer diagnosis to the date of death or to the study’s end date of 7/31/2023, whichever came first. Two patients had remote histories of stage I ovarian cancer and *in situ* fallopian tube cancer, respectively, prior to the current diagnosis of recurrence followed by platinum adjuvant chemotherapy. For them, the diagnosis date was determined to be the date of disease recurrence which was followed by adjuvant chemotherapy.

### 2.2 Genetic testing

Approximately 10ml of the patient’s peripheral whole blood was collected in an EDTA lavender top tube, provided in a kit by the test company, and sent to the test company by local transportation carrier within 72 hours. The physician chooses a test code or platform. In patients with known family history and identified genetic mutation, a single gene test was requested.

Tests done before 2015 were done only by Myriad genetics. In 2010, the test was called Comprehensive *BRACAnalysis*, which included *BRCA1* sequencing, and 5-site rearrangement panel, as well as *BRCA2* sequencing. After 2015, the test was called Integrated *BRACAnalysis*, which included comprehensive rearrangement testing in addition to the *BRCA1* and *BRCA2* sequencing. Only one test was performed by Ambry Genetics in 2017, which offered a *BRCA1/2* Analyses with *BRCAplus*-Expanded panel. According to the description, all genes are evaluated by NGS or Sanger sequencing of all coding domains, and well into the flanking 5’ and 3’ ends of all the introns and untranslated regions. More details on the sequencing methods is included in the **Supplementary Material**.

In most patients, peripheral blood was drawn and sent for germline testing, by one of the commercial germline testing companies, namely Myriad Genetics (Salt Lake City, UT), INVITAE (San Francisco, CA) and Ambry Genetics, (Aliso Viejo, CA). A patient is considered to have a pathogenic germline mutation if the test report shows the following: “positive result, pathogenic variant identified” (INVITAE), “positive result, clinically significant mutation identified” (Myriad), “positive for a deleterious mutation” (Myriad), or “Positive: pathogenic mutation detected) (Ambry).

### 2.3 Statistical analysis

Survival curves were plotted using Kaplan-Meier method. The descriptive statistics such as median survival time, with corresponding 95% confidence intervals were calculated based on the estimate of the survival function with the same method. The length of OS, PFS, and platinum free recurrence intervals between the HRD and HRP patient groups was obtained correspondingly. Log-rank test was conducted to compare the difference between patient groups.

We compared various patient characteristics between the HRD and HRP groups. We used Student’s t test to compare continuous data (age difference) and Fisher’s exact test for categorical variables. The level of statistical significance assumed in the analyses was 0.05. All statistical analyses were performed using R (version 4.3.2, R Foundation for Statistical Computing).

## 3 Results

### 3.1 Patient characteristics

Forty-four patients were eligible for this study, which included 21 in the HRD group and 23 in the HRP group. All tests were performed on the initial diagnosis specimen. HRD scores were obtained from Foundation Medicine, Inc, as a data transferring agreement in 31 patients; and 7 patients had commercial testing which was only qualitative of <16 (HRP) or ≥16 (HRD). Six patients were included in the HRD group based on their positive germline mutation, and in 5 of them, NGS tests were not performed.

The clinical characteristics of both groups are shown in **Table 1**. The median patient age was 58 in the HRD group and 61 in the HRP group (p=0.407). Fourteen (66.7%) and twenty-one (91.3%) patients had stage III/IV disease at presentation (p=0.064), respectively; with the remaining 9 patients presented with stage I or II disease but all experienced disease recurrence. Twenty (95.2%) and seventeen (73.9%) patients in the HRD and HRP groups, respectively, had serous carcinoma (p=0.097), while the minority of patients with mucinous (n=3) and clear cell (n=1) tumors all belonged to the HRP group.

**Table 1:** Patient characteristics.

	HRD (N=21)	HRP (N=23)	P value
Median age (year-old) Range	58 38-85	61 33-92	0.407
Stages			
IC + II IC II	7 (33.4%) 1 (4.8%) 6 (28.6%)	2 (8.6%) 1 (4.3%) 1 (4.3%)	0.417
III/IV	14 (66.7%)	21 (91.3%)	0.064
Histology Serous Non-Serous Clear cell Mucinous Adenocarcinoma	20 (95.2%) 1 (5.3%) 0 0 1 (5.3%)	17 (73.9%), 6 (26.0%) 1 (4.3%) 3 (13.0%) 2 (8.7%)	0.097
Debulking status (exclude stage I) Complete + optimal No Residual disease Optimal debulking Suboptimal debulking No surgery	N=20 18 (90.0%) 11 (55.0%) 7 (35.0%) 1 (5.0%) 1 (5.0%)	N=22 16 (72.7%) 9 (45.5%) 7 (31.8%) 1 (4.5%) 5 (22.7%)	0.243
Stage 3/4 optimal debulking	13 (61.9%)	15 (65.2%)	1
Neoadjuvant treatment	9 (42.9%)	4 (17.4%)	0.099
Platinum-based chemotherapy	21 (100%)	23 (100%)	N/A
Intraperitoneal chemotherapy HIPEC	9 (42.8%) 1 (4.8%)	7 (30.4%) 0	0.354
Use of PARP inhibitor in the subsequent lines, or maintenance therapy	7 (33.3%)	4 (17.4%)	0.303

HIPEC, Hyperthermic Intraperitoneal Chemotherapy; HRD, Homologous Recombination Deficiency; HRP, Homologous Recombination Proficiency; PARP, Poly (ADP‐ribose) Polymerase.

Most of the patients had optimal debulking, accounting for 90.0% of the HRD group and 72.7% of the HRP group (p=0.242). One patient in the HRD group and five patients in the HRP group did not receive surgery due to patient refusal. In addition, 42.9% of the HRD group and 17.4% of the HRP group had neoadjuvant chemotherapy (p=0.099). All patients completed platinum-based adjuvant chemotherapy following surgery. Sixteen patients also received IP chemotherapy, accounting for 42.8% of the HRD group and 30.4% of the HRP group (p=0.354). No patient received PARP inhibitor as maintenance in the first-line setting. Seven (33.3%) patients in the HRD group and 4 (17.4%) patients in the HRP group received PARP inhibitors as subsequent-line or maintenance therapy (p=0.303).

In the HRD group, 8 patients had germline mutations (**Tables 2**, **3**). This included 4 patients with *BRCA1*, one with *BRCA2*, one with *RAD51C*, one with *BRIP1*, and one with *CHEK2*. When performing NGS testing, there were 3 somatic *BRCA* 1/2 mutations (one of them also had germline mutation) and 3 somatic *BRIP1* mutations in the HRD group. *Tp53* mutation was present in 93.8% and 78.3% in the tested HRD and HRP groups, respectively (p=0.370) (**Table 2**). The lack of *Tp53* mutations were mainly detected in cases with mucinous and clear cell carcinomas.

**Table 2:** Next generation sequencing and genetic test results.

	HRD N=21	HRP N=23	P value
Germline mutation *BRCA 1* *BRCA 2* *RAD51C* *BRIP1* *CHEK2*	8 4 1 1 1 1	0 0 0 0 0 0	N/A
Tumor mutations constituting HRD *BRCA 1, 2* *BRIP1* *PTEN* *NF* *RAD51C* *KRAS* *PIK3CA* *RB1 loss* None *TP 53* mutation Yes No	N=16 3 3 2 2 1 0 0 0 2 15 (93.8%) 1 (serous)	N=23 0 0 1 0 0 2 2 1 13 18 (78.3%) 5 (3 mucinous, 1 clear cell, 1 serous)	N/A 0.370
Tumor mutation burden Low Intermediate	N=12 7 (58.3%) 5 (41.7%)	N=18 17 (94.4%) 1 (5.6%)	0.026
HRD LOH score Median Range	N=12 21.6 16.23-33.64	N=19 9 0.05-14.21	1.187x10^-7^

LOH, Loss of Heterozygosity; HRD, Homologous Recombination Deficiency; HRP, Homologous Recombination Proficiency.

**Table 3:** Characteristics of germline mutations.

	Serial number	Age at diagnosis	Mutation	HGVS nomenclature	Abnormality on the report	Test performed	LOH status	Test company	PARP inhibitor use
1	#25	68	*RAD51C*	NM_058216.3:c.709C>T (p.Arg237Ter)	c.709C>T (p.Arg237*)	Breast and gynecologic cancers guideline- based panel	High (18.74)	Invitae	Yes
2	#34	77	*BRCA1*	NM_007294.4:c.68_69del (p.Glu23fs)	c.68_69del (p.Glu23Valfs*17)	Integrated BRACAnalysis	High (33.64)	Myriad	No
3	#40	85	*BRIP1*	NM_032043.3:c.141del (p.Thr48fs)	c.141delC	Common Hereditary Cancers Panel	Not done	Invitae	No
4	#64	39	*BRCA1*	NM_007294.4:c.190T>G (p.Cys64Gly)	C64G(309T>G)	Single site analysis	Not done	Myriad	No
5	#65	54	*CHEK2*	NM_007194:c.902del (p.leu301fs)	c.902del	Common Hereditary Cancers Panel	Not done	Invitae	No
6	#66	58	*BRCA2*	NM_000059.4:c.1763_1766del (p.Asn588fs)	c.1763_1766delATAA	BRCA1/2 Analyses with BRCAplus-Expanded	Not done	Ambry	No
7	#67	43	*BRCA1*	NM_007294.4:c.3008_3009del (p.Asn1002_Phe1003insTer)	3127delTT	Comprehensive BRACAnalysis	Not done	Myriad	No
8	#68	38	*BRCA1*	NM_007294.4:c.68_69del (p.Glu23fs)	187delAG	Integrated BRACAnalysis	Not done	Myriad	No

HGVS, Human Genome Variation Society.

The test companies:

Myriad Genetics, Salt Lake City, UT

Invitae, San Francisco, CA

Ambry Genetics, Aliso Viejo, CA

The proportion of cases with intermediate TMB (6-15 Muts/Mb) was significantly higher in the HRD group (41.7%) compared to the HRP group (5.6%) (p=0.026). None of our patients had high TMB (**Table 2**).

The medians and ranges for LOH scores in the two groups were 21.6 (range, 16.23-33.64) for HRD patients and 9 (range, 0.05-14.21) for HRP patients (p =1.187x10-7) (**Table 2**). Interestingly, the one case of clear cell carcinoma had a LOH score of 0.05, and the median for the three cases of mucinous carcinoma was 5.23 (range, 4.69-6.16).

Six patients were included in the study based on positive germline mutations, *BRCA1* (n=3), *BRCA2* (n=1), *CHEK2* (n=1) and *BRIP1* (n=1). The genetic mutations and their LOH scores, the use of PARP inhibitors of the 8 patients with germline mutations are included in **Table 3**. The characteristics of the two additional patients with somatic, but no germline *BRCA* mutations are included in **Table 4**.

**Table 4:** Characteristics for patients with somatic BRCA mutations only.

	Serial number	Age at diagnosis	Gene mutation	Sequence on NGS testing	LOH status	Germline test	Test company	PARP inhibitor use
1	#7	60	BRCA 2	T1887fs*21	High (21.11)	Not detectable	Invitae	Yes
2	#36	75	BRCA 1	D214fs*20	High (>16)	Not detectable	Ambry	Yes

NGS, next generation sequencing; test company for both cases: Foundation Medicine, Inc. Cambridge, Massachusetts.

### 3.2 Patient outcomes

As of the study cutoff date of July 31, 2023, 39 out of 44 patients experienced disease progression, including 85.7% (18 out of 21) and 91% (21 out of 23) patients in the HRD and HRP groups, respectively. Of the three patients without recurrence in the HRD group, two patients had *BRCA* mutations. Furthermore, 9 (42.9%) patients in the HRD group and 7 (30.4%) patients in the HRP group were still alive. The median follow-up time for the entire cohort was 66.7 months (range 7.8-216.5 months).

**Figure 1:**
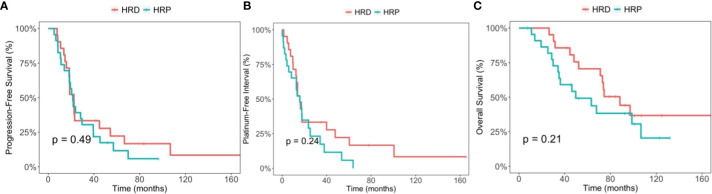
Comparison between the homologous recombination deficiency (HRD) group and the homologous recombination proficiency (HRP) group. **(A)** Kaplan-Meier estimates of progression-free survival. **(B)** Kaplan-Meier estimates of platinum-free interval. **(C)** Kaplan-Meier estimates of overall survival.

The median PFS was 22.5 months (95% CI, 18.5-66.6) in the HRD group and 21.5 months (95% CI, 18.3-39.5) in the HRP group (p=0.49). The median PFI was 15.8 months (95%CI, 12.4-60.4) and 15.9 months (95% CI, 8.3-34.1) (p=0.24), respectively. The median OS was 88.2 months (95% CI, 71.2-non applicable [NA]) in the HRD group, and 49.7 months (95% CI 35.1-NA) in the HRP group (p=0.21). Kaplan-Meier curves are displayed in **Figure 1**.

**Figure 2:**
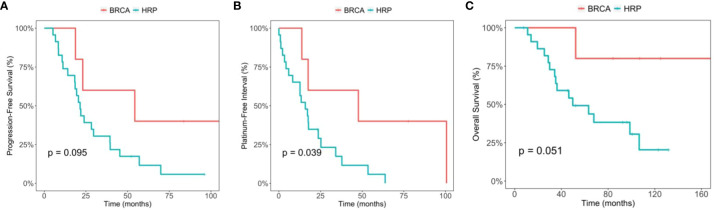
Comparison between the BRCA germline mutation group and the homologous recombination proficiency (HRP) group. **(A)** Kaplan-Meier estimates of progression-free survival. **(B)** Kaplan-Meier estimates of platinum-free interval. **(C)** Kaplan-Meier estimates of overall survival.

In the exploratory analysis, we compared the PFS and OS between the germline *BRCA* mutation group (5 patients), and the HRP group. PFS was 54.3 months (95% CI, 23.1-NA) and 21.5 months (95% CI, 18.3-39.5), respectively (p=0.095). The OS was not reached in the germline *BRCA* mutation group, and 49.7 months (95% CI 35.1-NA) in the HRP group (p=0.051) (**Figure 2**). Of note, 3 out of 5 patients from *BRCA* mutation positive group had recurrence, and none of them used PARP inhibitors after recurrence. When comparing the PFI between the *BRCA* mutation group and the HRP group, there was a statistically significant difference. PFI was 47.7 months (95%CI 17.6-NA) in the BRCA mutation group and 15.9 months (95% CI 8.3-34.4) in the HRP group (p=0.039).

**Figure 3:**
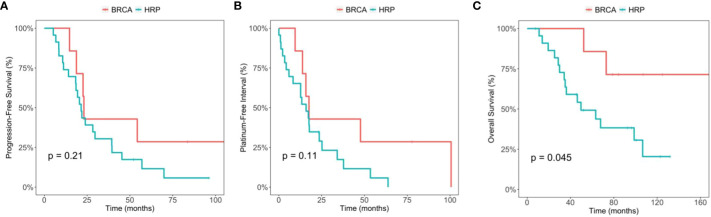
Comparison between the BRCA germline/somatic mutation group and the homologous recombination proficiency (HRP) group. **(A)** Kaplan-Meier estimates of progression-free survival. **(B)** Kaplan-Meier estimates of platinum-free interval. **(C)** Kaplan-Meier estimates of overall survival.

We then included two patients with somatic only *BRCA* mutations to the *BRCA* mutation positive group (n=7) (**Table 4**), and compared the PFS, PFI and OS with those of the HRP (**Figure 3**). In this analysis, the median OS is NA (95% CI 73, NA) for *BRCA* mutation group and 49.7 months (95% CI 35.1, NA) for HRP, respectively, which was statistically significant (p=0.045). The comparison in PFS and PFI did not show statistical significance (**Figure 3**). Of note, both patients developed recurrence and received PARP inhibitor treatment in the later lines.

In patients with any germline mutations including *BRCA* or other mutations (n=8), PFS was 38.7 months (95% CI 18.7-NA) and OS was not reached (95% CI 74.5 to NA), respectively. When comparing those with the HRP groups, neither of the PFS and OS differences was significant (**Supplementary Figure 2**). In addition, PFI was 32.7 months (95% CI 13.8-NA) in the all genetic mutation group, and 15.9 months (95% CI 8.3-34.1) in the HRP group, showing a strong trend toward statistical difference (p= 0.052).

**Figure 4:**
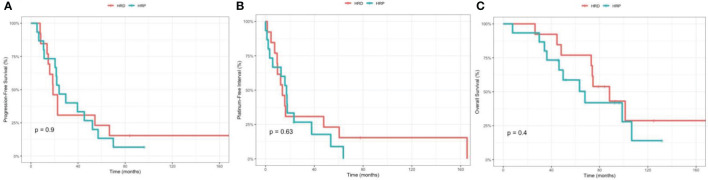
Comparison between the homologous recombination deficiency (HRD) group and the homologous recombination proficiency (HRP) group in stage III/IV, optimally debulked patients. **(A)** Kaplan-Meier estimates of progression-free survival. **(B)** Kaplan-Meier estimates of platinum-free interval. **(C)** Kaplan-Meier estimates of overall survival.

Among the 44 patients, 28 patients had stage III/IV disease that underwent optimal debulking surgery, including 13 patients with HRD and 15 patients with HRP status. Among the 28 patients, the median PFS was 18.7 months (95% CI 14.7, NA) for HRD and 23.9 months (95% CI 20.5, 57) for HRP (p=0.9), respectively. The median PFI was 13.8 months (95% CI 9.5, NA) for HRD and 17.2 months (95% CI 5.9, NA) for HRP (p=0.63), respectively. The median OS was 88.2 (95% CI 73, NA) for HRD and 67.8 months (95% CI 46.3, NA) for HRP (p=0.4), respectively (**Figure 4**).

If the LOH cut-off score was decreased to 14 as in the ARIEL 3 study, one patient would be added to the HRD group, and the difference in PFS, PFI and OS between the HRD and HRP groups was not no statistically significant (Data not shown).

## 4 Discussion

HRD status has been postulated as a predictive marker to indicate clinical benefits from PARP inhibitor maintenance therapy in platinum-sensitive ovarian cancer. In both first- and second-line settings, PARP inhibitor maintenance therapy has demonstrated improvement in PFS compared to placebo, in patients with HRD status and germline and somatic *BRCA* mutations, following initial response to platinum agents (23–25, 38–40). Previous studies reported that both germline and somatic mutations of HR genes predict platinum response and survival in advance stage ovarian cancers (31). Our study aimed to test if HRD predicts platinum response, investigating whether HRD status correlates with longer PFS or PFI following adjuvant platinum-based chemotherapy. Our results failed to demonstrate such a correlation. The median PFS was similar in both HRD and HRP groups (22.5 months vs 21.5 months, p=0.24), as well as the median PFI (15.8 months vs 15.9 months, p=0.49).

We compared our results with those previously reported in the literature. First, we examined PFS data from the control arm of the PAOLA trial, a randomized phase III study that used olaparib and bevacizumab as maintenance therapy following cytoreduction and adjuvant chemotherapy with bevacizumab in the first-line setting (41). Among those who received chemotherapy and bevacizumab with no PARP inhibitor maintenance treatment, the median PFS was 17.7 months in *BRCA* mutations carriers, 16.6 months in patients with HRD status, and 16.0 months in patients with HRP tumors, showing no significant difference. Second, we reviewed data from the PRIMA trial, a randomized phase III study that evaluated the effect of maintenance niraparib following cytoreduction and adjuvant chemotherapy in the first-line setting (24). Of note, this study enrolled patients with more adverse risk factors, particularly those with residual disease after cytoreduction. For those who only received adjuvant chemotherapy, the median PFS was 5.4 months in the HRP group, 10.9 months in the HRD group, and 8.2 months in *BRCA* wild-type HRD group. Although a numerically longer PFS was seen in the HRD group, no pre-planned statistical analysis was available to query whether this difference was statistically significant. Furthermore, GOG 218 performed further analysis on the impact of HR genes. GOG 218 was a randomized, phase III trial for patients with advanced ovarian cancer who received standard chemotherapy with carboplatin and paclitaxel and also extended course of bevacizumab in the study group (15). In that study, the HRD status was determined by mutations on a selected subset of genes predicted to impact HR repair, including *ATM, ATR, BARD1, BLM, BRCA1, BRCA2, BRIP1, CHEK2, MRE11A, BNB, PALB2, RAD51C, RAD51D, RBBP8, SLX4* and *XRCC2*. It showed significantly improved PFS as well as OS in BRCA mutation carriers as well as other HR genes (37). Therefore, whether HRD is a predictive marker for platinum sensitivity will still need further studies.

HRD status as a prognostic marker for estimation of OS has been presented in the final analysis of GOG 218 (15). Patients with *BRCA1/2* gene mutations or with other HR repair mutations had longer median OS (61.2 months and 56.2 months, respectively) compared to *BRCA* wild-type participants (42.1 months), with hazard ratios of 0.62 (95% CI 0.52-0.73) and 0.65 (95% CI 0.51-0.85), respectively (15). In our study, the median OS was 88.2 months (95% CI 73.0-NA) in the HRD group, and 49.7 months (95% CI 35.1-NA) in the HRP group, showing a trend toward longer survival but not statistically significant (p=0.21), likely related to small sample size. In addition, as the HRD group had lower percentage of patients with stage III/IV disease, this could be a bias toward better OS in this group. It should also be noted that in our study, 33.3% of the patients in the HRD group versus 17.4% of patients in the HRP group received subsequent-line or maintenance PARP inhibitor therapy after subsequent-line chemotherapy, which might contribute to the trend toward longer OS in HRD patients.

The *BRCA* mutation carriers in our study had longer OS (median OS not achieved) than the HRP group (49.7 months [95% CI 35.1-NA]), with a trend toward statistically significance (p=0.051). This result aligns with the findings from the GOG 218 study (15, 37). By including patients with somatic *BRCA1/2* mutations to the *BRCA* positive group, the OS difference was larger, and reached a statistical significance. One caveat is that both of the two patients with somatic BRCA mutations received PARP inhibitor treatment in later lines, possibly contributing to their longer OS. Nevertheless, this notion of longer survival in *BRCA* mutation carriers is supported by other larger observations as well. A large meta-analysis based on 26 studies conducted between 1987 and 2010 (34) showed that both *BRCA1* and *BRCA2* carriers had improved 5-year OS than non-carriers. In another study based on the cases included in The Cancer Genome Atlas project, observed between 2009 and 2010, a positive association between *BRCA2* but no *BRCA1* mutations and longer OS, longer platinum-free duration was revealed (35). Overall, it seems that the *BRCA* mutation carrier, and probably HRD status, may be a favorable prognostic marker for advanced-stage ovarian cancer.

In this study, we determined HRD status based on commercially available FoundationOne CDx Assay, and used the commercial report of HRD score ≥16 as a cut off. In order to compare our result to the LOH cutoff score of ≥14 used in the ARIEL2 study (23), we performed an exploratory analysis to apply a LOH cutoff score of ≥14, taking advantage of the cases where actual scores were reported through the research data exchange. Our exploratory analysis showed a similar result. Other commercially available platforms adopt slightly different criteria to determine the HRD status. For instance, Myriad’s myChoice CDx uses telomeric allelic imbalance and large-scale state transitions in addition to LOH to generate their own HRD score, which identifies HRD in about 48% of ovarian cancer patients (42). The myChoice test developed by Myriad Genetics was applied in the PRIMA study and the PAOLA study (24, 41). Had our study used a different platform, our HRD results and the resulting associations between HRD status and platinum sensitivity might have been altered to at least some degree. Of note, more HRD assays are under development, including functional assays, and academic tests (20).

There was numeric imbalance between the HRD and HRP groups in patient characteristics, such as patients with stage III/IV versus stage I/II, percentages of serous carcinoma and other histological subtypes, optimal and suboptimal debulkings, as well as patients receiving neoadjuvant treatments or not (**Table 1**), although none of those differences reached statistical significance. Our study is limited by its small sample size of 44 patients, which is related to the size of our clinical practice. Even if a true difference were to exist between HRD and HRP patients, its statistical significance could be masked by the heterogeneity of individual tumor history, IP treatment, and IV treatment variation in such a small patient population. In an attempt to compensate this heterogeneity, we adopted stricter selection criteria and conducted analysis to subgroups, such as narrowing down to the patients with stage III/IV and optimal debulking, while there was still no significant difference between HRD and HRP groups. In addition, we included patients diagnosed from 2010 and 2020, during which time the standard of care varied from universal adoption of IP chemotherapy, to no IP but neoadjuvant chemotherapy and to the use of maintenance therapy with PARP inhibitors. This difference could also affect the analysis of the prognostic and predictive role of HRD.

HRD status is similar to a term of “*BRCA*-ness” used earlier before the availability of HRD testing. “*BRCA*-ness” phenotype denotes a group of patients who carry germline or somatic mutations in genes that are involved in the HR pathway, or who possess epigenetic modification of the promoter regions of these genes (43, 44). In breast cancer literature, carboplatin doubled the objective response rate in *BRCA* mutated patients with metastatic breast cancer when compared with docetaxel (45). Carboplatin also increased pathological complete response rate when added to standard neoadjuvant chemotherapy in triple-negative breast cancer with a “*BRCA-*ness” phenotype (46, 47).

Our observations on the PFS and PFI between HRD versus HRP status in ovarian cancer did not entirely align with the current body of clinical evidence, which suggested a positive association between platinum responsiveness and the presence of either *BRCA* mutation or “*BRCA*-ness” phenotype in patients with ovarian and breast cancers. In our general analysis, HRD status alone did not have a predictive value for platinum responsive-ness. However, in our exploratory analysis, patients with *BRCA* mutations (n=5) had significantly longer PFI when compared with the HRP group (47.7 months [95%CI 17.6-NA] vs 15.9 months [95%CI 12.4-60.4], p=0.039). However, when the two patients with somatic *BRCA* mutations were added, the significance on PFI was not detected anymore. Since this study has very small number of patients in those subgroups, a signal of PFI difference is emerging, but caution should be exercised to draw a firm conclusion.

Our result prompts us to postulate that prolonged OS of the *BRCA* mutation carriers may be a result of their better response to platinum-based chemotherapy due to a stronger or deeper genomic scarring involved in DNA repair, which is crucial to repair the cytotoxic effect caused by platinum drugs. The other genes in HRD that are implicated in the DNA repair pathway may not reach such a strong impact to the genome.

The susceptibility to platinum agents could be fully- or partially-independent of the lethal mechanism of double stranded DNA damage. Another DNA repair process known as nucleotide excision repair (NER) was also described in the literature. NER pathway inactivation is associated with enhanced platinum sensitivity, similar to that seen in *BRCA 1/2* mutated tumors in ovarian cancers (48).

A higher percentage of HRD tumors in our study possessed intermediate TMB scores (defined as 6-15 Muts/Mb) compared to HRP tumors, while the majority of ovarian cancers have low TMB. High TMB is known to be associated with genomic alterations such as *POLE* and *PTEN* mutations, as well as *BRCA1* (49). There also appears to be an association between high TMB, DNA damage repair gene alterations, and improved prognosis (50). It has been postulated that synergistic therapeutic effects can be expected from the combination of PARP inhibitors and immunotherapy in patients with HRD, and preliminary results were promising (51).

In our study, more patients with high-grade serous carcinomas were in the HRD group, while those patients with mixed, mucinous, and clear-cell histologies were predominantly in the HRP group. The frequency of HRD in serous versus non-serous ovarian cancers has been studied with conflicting results. While Pennington et al. found similar rates among serous and non-serous tumors (31), Sugiono et al. reported reduced HRD frequencies in patients with clear cell (28%) and mucinous (16%) carcinomas compared to high-grade serous carcinomas (44%) (52). The most common mutations found among these non-serous subtypes were *ATM* mutations, followed by *BRCA2* mutations.

## 5 Conclusion

Our study demonstrated that there is no significant difference in PFS or in PFI in patients with HRD status who received platinum-based chemotherapy compared to those with HRP status. This indicates that HRD may not be a predictive marker for platinum response. *BRCA* mutation carriers may have a prolonged PFI, in comparison to the HRP group. HRD status was associated with a trend toward longer OS than in HRP, although statistically significance was not reached. Germline and somatic *BRCA* mutation carriers showed a statistically prolonged OS than the HRP group. A higher percentage of HRD tumors had intermediate TMB scores, while HRP tumors had predominantly low TMB scores.

## Data availability statement

The raw data supporting the conclusions of this article will be made available by the authors, without undue reservation.

## Ethics statement

The studies involving humans were approved by Maimonides Institutional Review Boards. The studies were conducted in accordance with the local legislation and institutional requirements. The participants provided their written informed consent to participate in this study.

## Author contributions

YX: Writing – review & editing, Writing – original draft. Y-JC: Writing – original draft. YW: Writing – review & editing, Formal analysis. AS: Writing – original draft, Data curation. AJ: Writing – original draft, Data curation. RB: Writing – original draft, Data curation. JS: Writing – original draft. JY: Writing – original draft. VK: Writing – original draft, Formal analysis.
