# Supplementary figures and images for: The prognostic and predictive value of homologous recombination deficiency status in patients with advanced stage epithelial ovarian carcinoma after first-line platinum-based chemotherapy
Source: Front Oncol. 2024 Jun 10;14:1372482. doi: 10.3389/fonc.2024.1372482 (PMC11194312; doi:10.3389/fonc.2024.1372482)

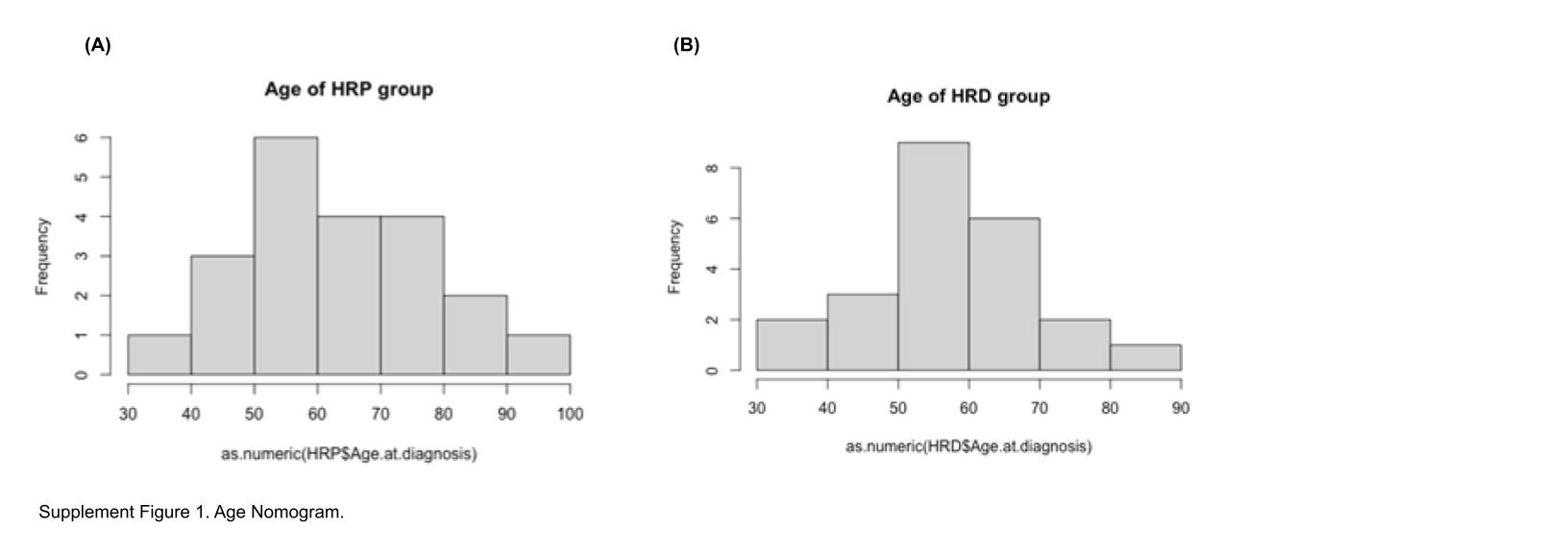

Supplement: Supplementary file 1 [file Image_1.jpeg]

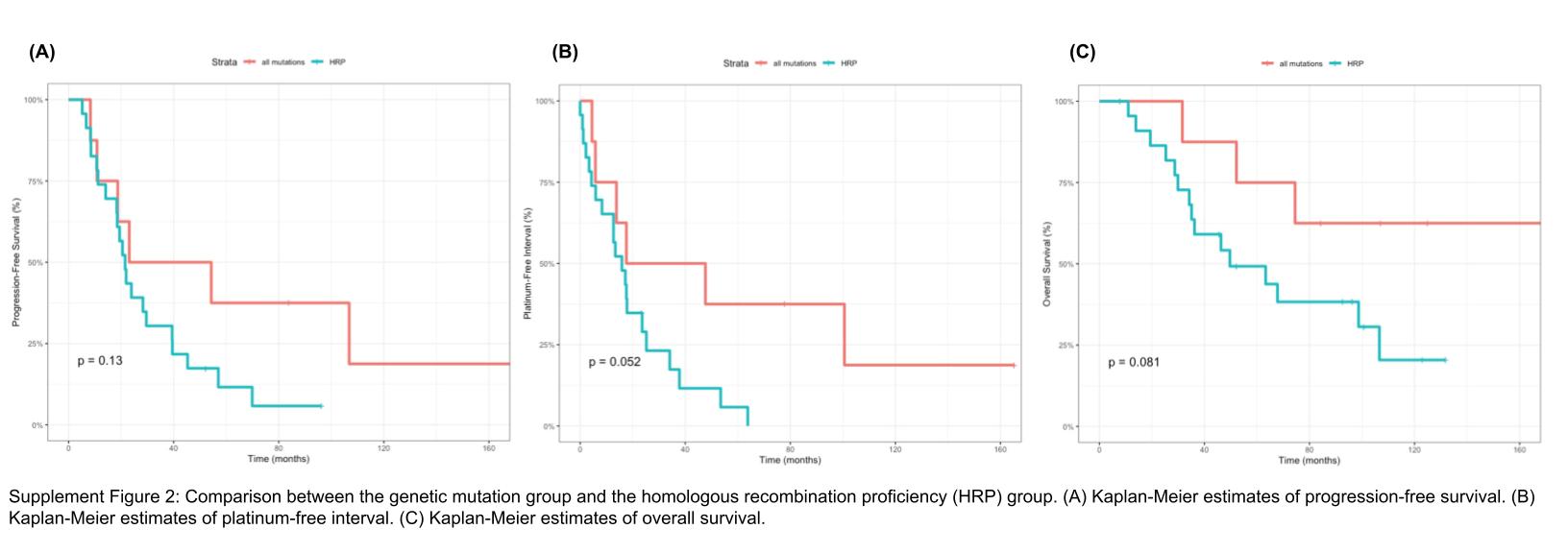

Supplement: Supplementary file 2 [file Image_2.jpeg]
