# Supplementary material for: The prognostic and predictive value of homologous recombination deficiency status in patients with advanced stage epithelial ovarian carcinoma after first-line platinum-based chemotherapy
Source: Front Oncol. 2024 Jun 10;14:1372482. doi: 10.3389/fonc.2024.1372482 (PMC11194312; doi:10.3389/fonc.2024.1372482)
Supplement: Supplementary file 3 [file DataSheet_1.docx]

**Supplemental Material**

**Methods**

Genetic testing

The *BRCA2*Portuguese founder mutation, c.156_157insAlu (also known as 384insAlu) is detected by NGS and confirmed by MLPA. A secondary sequencing method is performed for any regions with insufficient read depth coverage for reliable heterozygous variant detection. Potentially homozygous variants, variants in regions complicated by pseudogene interference, and variant calls not satisfying depth of coverage and variant allele frequency quality thresholds are verified by Sanger sequencing. Gross deletion/duplication analysis is performed for the covered exons and untranslated regions of all sequenced genes using read-depth data from NGS data with confirmatory multiplex ligation-dependent probe amplification (MLPA) and/or targeted chromosomal microarray.

After 2018, three tests were performed by INVITAE on their multigene panel. Sequence analysis and deletion/duplication testing of 46 genes included as a common hereditary cancers panel were used for 2 patients, and a breast and gynecologic cancers guideline based panel was used for 1 patient. INVITAE uses next-generation sequencing technology.

INVITAE is a College of American Pathologists-accredited and Clinical Laboratory Improvement Amendments-certified clinical diagnostic laboratory performing full-gene sequencing and deletion/duplication analysis using NGS technology.

The sequence analysis covers clinically important regions of each gene, including coding exons and 10 to 20 base pairs of adjacent intronic sequence on either side of the coding exons in the transcript listed below, depending on the specific gene or test. In addition, the analysis covers select non-coding variants. Any variants that fall outside these regions are not analyzed. Any limitations in the analysis of these genes will be listed on the report.

Homologous Recombination Deficiency status

The positive homologous recombination deficiency (HRD) status is defined as tBRCA-positive and/or LOH (loss of Heterozygosity) high. The computation of the cut-off percent to define a high genomic LOH was developed in conjunction with the ARIEL 2 where that LOH high was associated with improved progression-free survival (PFS) from Rubraca (rucaparib) maintenance therapy (1, 2). While the cut-off selected in the study was LOH ≥14, the approved commercial test settled on the LOH score ≥ 16 to be HRD positive.

To compute the percent genomic LOH for each tumor, LOH segments were inferred across the 22 autosomal chromosomes using the genome-wide aneuploidy/copy number profile and minor allele frequencies of the more than 3500 polymorphic SNPs sequenced in the Foundation Medicine’s NGS-based T5a assay. Briefly, a comparative genomic hybridization (ie, log-ratio profile of the sample) was obtained from the NGS sequencing data by normalizing the sequence coverage obtained at all exons and genome-wide SNPs against a process-matched normal control. This profile was segmented and interpreted using allele frequencies of sequenced SNPs to estimate copy number (*Ci*) and minor allele count (*Mi*) at each segment (*i*). A segment was determined to have LOH if *Ci ≠ 0* and *Mi = 0*.

For each tumor, the percent genomic LOH was computed as 100 times the total length of non-excluded LOH regions (*xi*) divided by the total length of non-excluded regions of the genome. In equation form:

∑_i_ x_i_

Percent genomic LOH = 100 x -----------------------------------

L genome – L exclusions

Where

x_i_ : Length of eligible LOH at segment *i*

L genome: Total length of genome with SNP coverage, which is 2·78×109 base pairs

L exclusions: Total length of genome excluded for LOH analysis

**References:**

1. Swisher EM, Lin KK, Oza AM, Scott CL, Giordano H, Sun J, et al. Rucaparib in relapsed, platinum-sensitive high-grade ovarian carcinoma (ARIEL2 Part 1): an international, multicentre, open-label, phase 2 trial. Lancet Oncol. 2017;18(1):75-87.

2. Medicine F. FoundationOne®CDx Technical Information: Foundation Medicine; [Available from: <https://www.foundationmedicine.com/sites/default/files/media/documents/2023-11/RAL-0003-24%20F1CDx%20Technical%20Label%20%28P170019_S048%29_Clean.pdf>.
