# Supplementary material for: The prognostic and predictive value of homologous recombination deficiency status in patients with advanced stage epithelial ovarian carcinoma after first-line platinum-based chemotherapy
Source: Front Oncol. 2024 Jun 10;14:1372482. doi: 10.3389/fonc.2024.1372482 (PMC11194312; doi:10.3389/fonc.2024.1372482)
Supplement: Supplementary file 4 [file DataSheet_2.pdf]

FoundationOne CDx is designed to include genes known to be somatically altered in human solid tumors that are validated targets for therapy, either approved or in clinical trials, and/or that are unambiguous drivers of oncogenesis based on current knowledge. The current assay interrogates 324 genes as well as introns of 36 genes involved in rearrangements. The assay will be updated periodically to reflect new knowledge about cancer biology.

DNA GENE LIST: ENTIRE CODING SEQUENCE FOR THE DETECTION OF BASE SUBSTITUTIONS, INSERTION/DELETIONS, AND COPY NUMBER ALTERATIONS

ABL1	*ACVR1B*	*AKT1*	*AKT2*	*AKT3*	*ALK*	*ALOX12B*	*AMER1 (FAM123B)*	*APC*
AR	*ARAF*	*ARFRP1*	*ARID1A*	*ASXL1*	*ATM*	*ATR*	*ATRX*	*AURKA*
AURKB	*AXIN1*	*AXL*	*BAP1*	*BARD1*	*BCL2*	*BCL2L1*	*BCL2L2*	*BCL6*
BCOR	*BCORL1*	*BRAF*	*BRCA1*	*BRCA2*	*BRD4*	*BRIP1*	*BTG1*	*BTG2*
BTK	*C11orf30 (EMSY)*	*C17orf39 (GID4)*	*CALR*	*CARD11*	*CASP8*	*CBFB*	*CBL*	*CCND1*
CCND2	*CCND3*	*CCNE1*	*CD22*	*CD274 (PD-L1)*	*CD70*	*CD79A*	*CD79B*	*CDC73*
CDH1	*CDK12*	*CDK4*	*CDK6*	*CDK8*	*CDKN1A*	*CDKN1B*	*CDKN2A*	*CDKN2B*
CDKN2C	*CEBPA*	*CHEK1*	*CHEK2*	*CIC*	*CREBBP*	*CRKL*	*CSF1R*	*CSF3R*
CTCF	*CTNNA1*	*CTNNB1*	*CUL3*	*CUL4A*	*CXCR4*	*CYP17A1*	*DAXX*	*DDR1*
DDR2	*DIS3*	*DNMT3A*	*DOT1L*	*EED*	*EGFR*	*EP300*	*EPHA3*	*EPHB1*
EPHB4	*ERBB2*	*ERBB3*	*ERBB4*	*ERCC4*	*ERG*	*ERRFI1*	*ESR1*	*EZH2*
FAM46C	*FANCA*	*FANCC*	*FANCG*	*FANCL*	*FAS*	*FBXW7*	*FGF10*	*FGF12*
FGF14	*FGF19*	*FGF23*	*FGF3*	*FGF4*	*FGF6*	*FGFR1*	*FGFR2*	*FGFR3*
FGFR4	*FH*	*FLCN*	*FLT1*	*FLT3*	*FOXL2*	*FUBP1*	*GABRA6*	*GATA3*
GATA4	*GATA6*	*GNA11*	*GNA13*	*GNAQ*	*GNAS*	*GRM3*	*GSK3B*	*H3F3A*
HDAC1	*HGF*	*HNF1A*	*HRAS*	*HSD3B1*	*ID3*	*IDH1*	*IDH2*	*IGF1R*
IKBKE	*IKZF1*	*INPP4B*	*IRF2*	*IRF4*	*IRS2*	*JAK1*	*JAK2*	*JAK3*
JUN	*KDM5A*	*KDM5C*	*KDM6A*	*KDR*	*KEAP1*	*KEL*	*KIT*	*KLHL6*
KMT2A (MLL)	*KMT2D (MLL2)*	*KRAS*	*LTK*	*LYN*	*MAF*	*MAP2K1 (MEK1)*	*MAP2K2 (MEK2)*	*MAP2K4*
MAP3K1	*MAP3K13*	*MAPK1*	*MCL1*	*MDM2*	*MDM4*	*MED12*	*MEF2B*	*MEN1*
MERTK	*MET*	*MITF*	*MKNK1*	*MLH1*	*MPL*	*MRE11A*	*MSH2*	*MSH3*
MSH6	*MST1R*	*MTAP*	*MTOR*	*MUTYH*	*MYC*	*MYCL (MYCL1)*	*MYCN*	*MYD88*
NBN	*NF1*	*NF2*	*NFE2L2*	*NFKBIA*	*NKX2-1*	*NOTCH1*	*NOTCH2*	*NOTCH3*
NPM1	*NRAS*	*NSD3 (WHSC1L1)*	*NT5C2*	*NTRK1*	*NTRK2*	*NTRK3*	*P2RY8*	*PALB2*
PARK2	*PARP1*	*PARP2*	*PARP3*	*PAX5*	*PBRM1*	*PDCD1 (PD-1)*	*PDCD1LG2 (PD-L2)*	*PDGFRA*
PDGFRB	*PDK1*	*PIK3C2B*	*PIK3C2G*	*PIK3CA*	*PIK3CB*	*PIK3R1*	*PIM1*	*PMS2*
POLD1	*POLE*	*PPARG*	*PPP2R1A*	*PPP2R2A*	*PRDM1*	*PRKAR1A*	*PRKCI*	*PTCH1*
PTEN	*PTPN11*	*PTPRO*	*QKI*	*RAC1*	*RAD21*	*RAD51*	*RAD51B*	*RAD51C*
RAD51D	*RAD52*	*RAD54L*	*RAF1*	*RARA*	*RB1*	*RBM10*	*REL*	*RET*
RICTOR	*RNF43*	*ROS1*	*RPTOR*	*SDHA*	*SDHB*	*SDHC*	*SDHD*	*SETD2*
SF3B1	*SGK1*	*SMAD2*	*SMAD4*	*SMARCA4*	*SMARCB1*	*SMO*	*SNCAIP*	*SOCS1*
SOX2	*SOX9*	*SPEN*	*SPOP*	*SRC*	*STAG2*	*STAT3*	*STK11*	*SUFU*
SYK	*TBX3*	*TEK*	*TET2*	*TGFBR2*	*TIPARP*	*TNFAIP3*	*TNFRSF14*	*TP53*
TSC1	*TSC2*	*TYRO3*	*U2AF1*	*VEGFA*	*VHL*	*WHSC1*	*WT1*	*XPO1*
XRCC2	*ZNF217*	*ZNF703*						

DNA GENE LIST: FOR THE DETECTION OF SELECT REARRANGEMENTS

ALK	*BCL2*	*BCR*	*BRAF*	*BRCA1*	*BRCA2*	*CD74*	*EGFR*	*ETV4*
ETV5	*ETV6*	*EWSR1*	*EZR*	*FGFR1*	*FGFR2*	*FGFR3*	*KIT*	*KMT2A (MLL)*
MSH2	*MYB*	*MYC*	*NOTCH2*	*NTRK1*	*NTRK2*	*NUTM1*	*PDGFRA*	*RAF1*
RARA	*RET*	*ROS1*	*RSPO2*	*SDC4*	*SLC34A2*	*TERC**	*TERT***	*TMPRSS2*

*TERC is an NCRNA

**Promoter region of TERT is interrogated

ADDITIONAL ASSAYS: FOR THE DETECTION OF SELECT CANCER BIOMARKERS

Loss of Heterozygosity (LOH) score
Microsatellite (MS) status
Tumor Mutational Burden (TMB)

SAMPLE
